# Neuraminidase Inhibitors and Hospital Mortality in British Patients with H1N1 Influenza A: A Re-Analysis of Observational Data

**DOI:** 10.1371/journal.pone.0160430

**Published:** 2016-09-01

**Authors:** Martin Wolkewitz, Martin Schumacher

**Affiliations:** Center for Medical Biometry and Medical Informatics, Institute for Medical Biometry and Statistics, Medical Center University of Freiburg, Freiburg, Germany; Hokkaido University Graduate School of Medicine, JAPAN

## Abstract

**Background:**

Observational studies claimed reducing effects of neuraminidase inhibitors (NI) on hospital mortality in patients with H1N1 influenza A. It has been criticized that such findings are prone to common and serious survival biases.

**Methods:**

With observational data from the FLU-CIN study group, multi-state and dynamic prediction models have been used to avoid such biases. The data included 1391 patients with confirmed pandemic influenza A/H1N1 infection collected during 2009-2010 in the UK. Due to their close relationship, the main outcome measures were hospital death and length of hospital stay.

**Findings:**

There is no direct effect of NI on the hospital death rate; the hazard ratio (HR) of NI was 1.03 (95%-CI: 0.64–1.66). The discharge rate is increased for NI patients (HR = 1.89 (95%-CI: 1.65–2.16)) indicating that NI-treated patients stay shorter in hospital than NI-untreated patients, on average 3.10 days (95%-CI: 2.07–4.14). We also showed that the initiation timing of NI treatment (≤ 2 days versus > 2 days after onset) made no difference on the effects on the hospital death and discharge hazards. The hazard ratios remain stable after adjusting for potential confounders measured at admission (such as comorbidities and influenza-related clinical symptoms).

**Conclusions:**

The potential beneficial effect of NI on hospitalized patients in the UK is rather a reduction of the length of hospital stay than a reduction of the mortality rate. There seems to be no confounding by indication and no differences if NI is given early or late. Different effects could be present in other populations (such as non-hospitalized individuals) or countries. Careful interpretation of the effect on length of hospital stay is needed due to potentially different discharge policies of NI-treated and NI-untreated patients.

## Introduction

In recent years, the influenza drug Oseltamivir, which is a neuraminidase inhibitor (NI) and marketed under the trade name Tamiflu, attracted considerable attention, after it was stockpiled extensively by multiple governments to prepare for upcoming pandemics. The BMJ have launched the Tamiflu campaign (bmj.com/tamiflu) to increase transparency, re-analyse clinical data, discuss clinical trials with real-world data and inform policy makers. Also The Lancet recently called for better research regarding NI for influenza [1].

Using randomised controlled trials (RCTs), two large meta-analyses from members of the Cochrane collaboration found that the drug had very limited clinical effects on complications and viral transmission [2] and reduced the duration of symptoms by only about half a day [3]. Also other researchers found only marginal treatment benefits in a meta-analysis of RCTs [4].

It has been argued that such RCTs usually include only patients without a real clinical need [5] and they were not designed or powered to give results regarding serious complications, hospitalization and mortality [6]. In contrast, several observational hospital studies -which usually include people who might really require treatment- found that the drug had a strong impact on mortality [7–10], especially for patients who started NI treatment within 2 days after illness onset [11]. In particular, the large meta-analysis of observational studies with 29.234 patients by Muthuri and colleagues, and this has stirred up the current controversial debate about the treatment effect [10].

This discrepancy could partly be explained by heterogeneity between RCTs (individuals with lower clinical need) and observational studies (individuals with higher clinical need) but also by several types of bias which frequently occur in observational studies and survival data [12–16]. Even though several groups of scientists challenged the results and the underlying statistical analysis [5, 17–20], it is still an open question whether the observational findings are subject to common survival biases. For instance, Jones et al claimed that the observational results are subject to time-dependent bias, which occurs if the time-dependent treatment is statistically considered as time-fixed [17, 18]. This type of bias is common in non-randomized treatment studies [21] and can lead to serious flawed findings in other cohort studies; for instance, the seemingly beneficial effect of skin cancer on survival [22, 23].

The observational results are also prone to a competing risk bias when using hospital data [19]. Classical survival techniques assume that discharged patients have the same mortality as hospitalized patients; an assumption which often does not hold: survival is usually improved after discharge [24]. Competing risk bias is common and can lead to unreliable findings [25].

Observational studies which retrospectively recruit patients on admission to hospital introduce selection bias as they do not observe those who are not admitted. This immortal time between influenza onset and hospital admission has to be addressed in observational analyses. Otherwise, length bias occurs if one assumes that patients are observed already from onset [13]. By distinguishing length, time-dependent and competing risk bias, we address the general issue of ‘survivorship bias’ which has been discussed by Freemantle and colleagues when analysing observational NI data [12]. All these three are common in medical research and can easily result in misleading conclusions [13, 14]. The impact of these types of bias is explored in our statistical paper for nosocomial infections [16].

In this article we use the observational FLU-CIN data [26] which is the British contribution of the meta-analysis from Muthuri et al. [10]. We perform a re-analysis which accounts for the time-dependent dynamics of this observational data and thus avoids all survival biases mentioned above. To control for confounding, regression techniques as well as a time-dependent propensity score approach are used.

## Methods

### Ethical approval

An ethics committee approval was not required in accordance with German law as the study is completely based on published data and patient information was anonymized and de-identified prior to analysis. According to Myles et al Thorax 2012;67:709–717: ‘Before commencement, FLU-CIN procedures were reviewed by the Ethics and Confidentiality Committee of the National Information Governance Board for Health and Social Care in England and approved for collection, storage and use of personal data for surveillance purposes.’

### The FLU-CIN data from the UK

The FLU-CIN data are described in detail by Myles et al [26]. In summary, the data are based on 13 sentinel hospitals situated in Nottingham, Leicester, London, Sheffield and Liverpool, with contributions from a further 45 non-sentinel hospitals in England and 17 in Scotland, Wales and Northern Ireland [26]. Data were obtained on 1520 patients with confirmed pandemic influenza A/H1N1 2009 infection. After excluding patients with implausible values or missing admission/discharge dates, 1391 patients entered our analysis. Missing values for the influenza onset date (n = 361) were imputed by regression techniques (see details in S1 File). The data (such as basic patient characteristics, influenza-related symptoms and crude outcomes) are described in detail in Table 1. An ethics committee approval was not required in accordance with German law as the study is completely based on published data and patient information was anonymized and de-identified prior to analysis.

**Table 1:** Description of the FLU-CIN data used for re-analysis. Following variables were used for confounding adjustment. All variables are measured at the time of hospital admission.

	number of patients (percentage) for binary or median (1st and 3st quartile) for continuous variables
**Basic characteristics**	
number of patients with H1N1 influenza A	1391 (100%)
gender (female)	800 (52.6%)
age <16 years	442 (31.8%)
age 16–64 years	867 (62.3%)
age >64 years	82 (5.9%)
**Comorbidities / conditions**	
obese	49 (3.2%)
asthma	385 (25.3%)
chronic obstructive	84 (5.5%)
pulmonary disease	
other lung disease	477 (31.4%)
heart disease	191 (12.6%)
renal disease	45 (3%)
liver disease	25 (1.6%)
cerebrovascular disease	123 (8.1%)
neurological disease	87 (5.7%)
diabetes	102 (6.7%)
immunosuppression	42 (2.8%)
pregnant	83 (6%)
anorexia	131 (8.6%)
arthralgia	96 (6.3%)
**Symptoms related to influenza measured at admission**	
chills	126 (8.3%)
coryza	265 (17.4%)
productive cough	606 (40%)
dry cough	585 (38.5%)
diarrhoea	201 (13.2%)
dyspnoea	575 (37.8%)
fever	1203 (79.1%)
headache	388 (25.5%)
malaise	291 (19.1%)
myalgia	309 (20.3%)
nausea	137 (9%)
rash	39 (2.6%)
seizures	32 (2.1%)
sore throat	378 (24.9%)
vomiting	447 (29.4%)
CURB-65 score = 0	454 (32.6%)
CURB-65 score = 1	560 (40.3%)
CURB-65 score = 2	320 (23.0%)
CURB-65 score = 3 or 4	57 (4.1%)
**Other characteristics**	
days between onset and admission	median (Q1,Q3) = 3 (1,5)
first wave: to 31 August 2009	545 (39.2%)
second wave: from 1 September 2009	846 (60.8%)
patient-days NI-untreated	3170 hospital days
patient-days NI-treated	5765 hospital days
patients who eventually	1028 (73.9%)
received NI	
patients who received	103 (7.4%)
NI before admission	
length of hospital	median (Q1,Q3) = 3 (2,7)
stay in days	mean (standard deviation) = 6.4 (9.5)
patients died in hospital	80 (5.8%)

**Fig 1:**
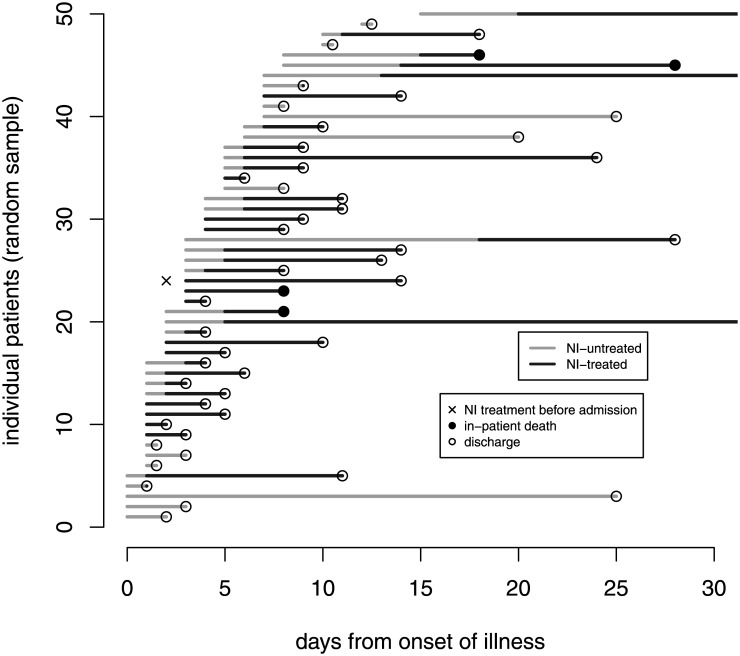
Graphical time-dependent display of 50 randomly selected patients. Each line shows one observed patient; sorted by the time to admission. To display the time-dependency of NI-treatment, NI-untreated time are marked in grey and NI-treated time is marked in black. Patients who reported NI treatment before hospital admission are marked with an ‘x’ and enter the hospital NI-treated (black). The corresponding clinical outcome event is marked with a filled circle (death) or a transparent circle (discharge).

The time-dependent dynamics are displayed with a random sample of 50 patients in Fig 1 and demonstrate the following issues. First, patients might take NI before admission. Second, there is some time between onset and admission. Third, NI intake depends on time from onset and finally, the observation ends with discharge or in-patient death. See also the risk sets Fig A in S1 File in the supplement.

### Statistical methods

We used multi-state and landmark methods to compare hospital mortality in individuals with H1N1 influenza who received neuraminidase inhibitors (NI) to those who did not receive NI.

#### Multi-state approach

To account for this time-dependent dynamics of the data (delayed entry, time-dependent NI intake and discharge as the end of follow-up), we use a multi-state model with following states: hospital admission, NI treatment, discharge and hospital death (see Fig B in S1 File). Time origin is the day on influenza onset, hence, time between onset and admission is addressed via external left-truncation. We calculated cumulative hazards to study the effect of NI on the death as well as discharge hazard by accounting for: 1) delayed entry due to admission, 2) treating NI as inter-mediate event and 3) in-hospital death and discharge as competing events (event-specific analysis) [27]. Hazard ratios for in-patient death and discharge were calculated in two time origins (time from onset and time from admission).

#### Length of hospital stay in days

A simplified multi-state model was used to estimate the effect of NI on the length of hospital in days. To do this, we used time of admission as time origin and considered the composite endpoint of in-hospital death and discharge; NI was treated as an inter-mediate event [28]. We performed stratified analyses for age group, hospital, time from onset to admission and CURB-65 score.

#### Landmark approach for dynamic prediction of in-hospital mortality

We used the landmark method [29] and chose landmark points between 2 and 10 days after onset after inspection of the corresponding risk sets (see Fig A in S1 File). The time frame is set to 20-days after landmark day. We calculated crude in-patient death probabilities up to 20 days after landmark time to make absolute dynamic predictions per landmark. For each landmark point, we considered only the patients who were hospitalized and at-risk to die or to be discharged. Then, we calculated death subdistribution hazard ratios using the Fine&Gray methodology [30, 31]; these subdistribution hazard ratios compare the cumulative risk to die in hospital up to 20-days after landmark day. Note that in contrast to the multi-state approach, patients remain in the treatment group from landmark point until discharge or death within 20-days; however, the NI-treatment assignment is updated on every landmark point anew.

#### Confounder adjustment

To identify potential confounding by indication, a Fine&Gray regression model was used to study factors associated with the cumulative probability of receiving NI in hospital. Confounders by indication were selected by backward selection and 5% significance level for entering effects. Further, confounder adjustment was made for the multi-state as well for the landmark approach. In the multi-state approach, we performed Cox regression models accounting for time-dependent covariates and used admission as time origin since all potential confounders were collected at the time of admission. To account for the most important factors, variable selection is made by backward selection and 5% significance level for entering effects (NI always included). In the landmark approach, we calculated the propensity score (based on the variables in Table 1) to receive NI on that landmark point. Then, we performed analyses adjusted via the inverse probability of treatment weighting using the propensity score (PS) [32] to get PS-adjusted death and discharge hazard ratios as well as death subdistribution hazard ratios.

## Results

**Fig 2:**
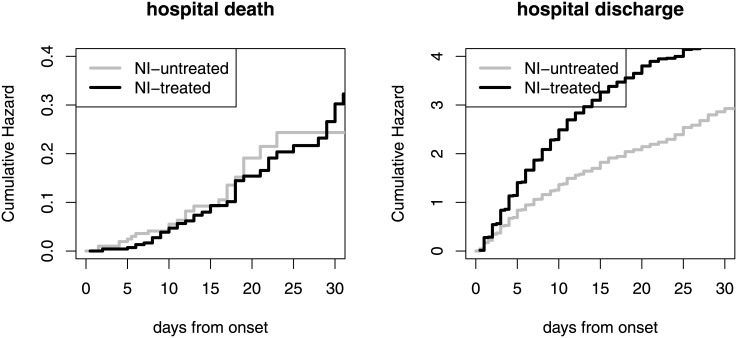
Cumulative hospital death and discharge hazards based on the Nelson-Aalen estimator; separately for NI-treatment. Delayed entry (external left-truncation), time-dependency of NI-treatment is accounted for. Additional plot with range up to 60 days is available in the supplement (Fig C in S1 File).

The cumulative hazards for hospital death and discharge are displayed in Fig 2. The corresponding unadjusted hazard ratios (NI-treated vs. NI-untreated) are HR = 1.03 (95%-CI: 0.64–1.66) for hospital death and HR = 1.89 (95%-CI: 1.65–2.16) for hospital discharge. This means that the *daily* risk to die in hospital is similar for NI-treated and NI-untreated patients (HR(death) = 1.03). But there is an effect on the hospital discharge hazard (HR(discharge) = 1.89) indicating that NI-treated patients stay shorter in hospital than NI-untreated patients. These hazard ratios do not basically change when switching the time origin from onset to admission: 1.03 (95%-CI: 0.63–1.67) for hospital death and 2.02 (95%-CI: 1.76–2.31) for discharge.

The increased discharge hazard of NI patients means that NI-treated patients stay shorter in hospital, on average 3.10 days (95%-CI: 2.07,4.14), see Table 2 and also the expected length of stay as a function of time (Fig E in S1 File). This reduction is higher for patients older than 65 years (about 5 days) and lower for the children (about 2 days). The effect of NI on length of stay also differs across hospitals ranging between 0.75 up to 5.16 days (Table 2). It is also more pronounced for patients with CURB score of 1 and 2 compared to score 0. But the effect changes the sign for patients with CURB score of 3 or 4 who are associated with a high mortality. A post-hoc subgroup competing risk analysis showed that among those high-risk patients (n = 57 patients of whom 10 died), NI is not associated with the discharge rate (HR = 1.06 (95%-CI: 0.51–2.21)) but NI seems to be associated with an decreased hospital death rate (HR = 0.29 (95%-CI: 0.08–1.09)), however not significantly.

**Table 2:** Reduction in length of hospital stay in days associated with neuraminidase inhibitors (* hospital center E is a composite center which contains patients from several hospitals with low contributions).

Variable	reduction in length of hospital stay, in days (95%-CI)
time from onset to admission	
0–2 days (n = 691)	2.65 (0.96,4.34)
2.1–4 days (n = 289)	2.19 (0.65,3.73)
4.1–7 days (n = 241)	4.79 (2.18,7.40)
>7 days (n = 170)	3.21 (0.65,5.78)
age group	
<16 years	1.78 (-0.54,4.11)
16–64 years	3.55 (2.43,4.67)
>64 years	4.99 (0.36,9.62)
hospital center	
A (n = 425)	3.92 (2.30,5.69)
B (n = 91)	2.79 (-2.30,7.88)
C (n = 146)	1.90 (-0.57,3.23)
D (n = 78)	1.03 (-0.74,2.81)
E* (n = 289)	2.64 (0.89,4.38)
F (n = 124)	0.75 (0.01,2.64)
G (n = 238)	5.16 (1.61,8.70)
CURB-65 score	
0 (n = 454)	1.84 (0.81,2.86)
1 (n = 560)	3.54 (1.60,5.47)
2 (n = 320)	4.23 (2.10,6.35)
3-4 (n = 57)	-2.11(-5.04,0.82)
overall	3.10 (2.07,4.14)

**Fig 3:**
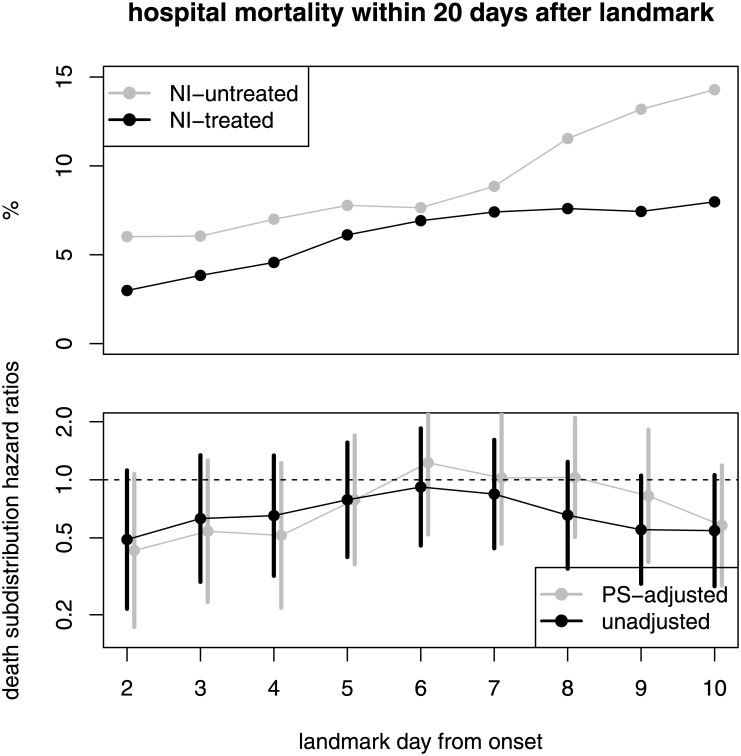
Upper panel: crude predicted in-hospital mortality within 30-days after landmark day; separately for NI-treatment. Lower panel: unadjusted and adjusted in-hospital death subdistribution ratios (NI-treated vs. NI-untreated) with 95-% CI (black).

The crude predicted hospital mortality within 20-days after landmark day is displayed in Fig 3; grouped by NI-treatment. Here, we observe a typical competing event phenomena in hospital data since the reduced length of stay for NI-treated patients has an indirect implication on the cumulative risk of dying in the hospital. Even though the death hazards of NI-treated and NI-untreated is similar (HR(death) is about 1), the 20-days hospital mortalities of the NI-treated are (non-significantly) reduced for almost all landmark days. This phenomena can be explained as follows. The crude daily risk to die in hospital is about 1% (55 / 5765 patient-days) for NI-treated and about 0.8% (25 / 3170 patient-days) for NI-untreated patients. Due to their reduced length of stay, NI-treated patients experience this daily risk for less days in hospital than NI-untreated patients. This is the reason why we observe less NI-treated patients dying in hospital which is also seen by comparing the crude hospital mortality between those patients who eventually and those who never received NI in hospital: 5.4% (55 / 1028 patients) versus 6.9% (25 / 363 patients).

### Confounder adjustment

Children and pregnant women were associated with a lower probability of receiving NI in hospital whereas following factors have a moderate increasing effect: male, other lung diseases, 1st wave, arthralgia, fever, dry cough, productive cough and headache. The probability of receiving NI varied across hospitals (see details in Table A in S1 File). There is no confounding by indication since the CURB-65 score as the main predictor for mortality is not associated with the probability of receiving NI.

After adjusting for potential confounding factors, the hazard ratios from the multi-state model remained stable (using admission as time origin): the adjusted hazard ratio for in-patient death was 1.04 (95-% CI: 0.62–1.75) and for discharge 2.00 (95-% CI: 1.73–2.31). Diarrhoea and higher CURB-65 score were associated with an increased whereas dry cough and myalgia with a lower hospital death hazard. There were several moderate factors associated with the hospital discharge hazard (Table D in S1 File).

Also the propensity score analysis provided the same picture: the hazard and subdistribution hazard ratios of interest were not affected by confounders (Fig 3). All PS-adjusted results are shown in the supplement of this paper (Fig D in S1 File).

### Early and late NI treatment

We also studied a potential effect of the timing of NI treatment by differentiating early (≤ 2 days after onset) (n = 460) and late (> 2 days after onset) (n = 568) NI treatment in the multi-state model; reference is no NI-treatment. The adjusted hazard ratio for hospital death was HR = 0.96 (95-% CI: 0.55–1.67) for early treatment and HR = 1.16 (95-% CI: 0.59–2.29) for late treatment. For hospital discharge, the adjusted hazard ratio was HR = 1.84 (95-% CI: 1.58–2.14) for early treatment and HR = 1.72 (95-% CI: 1.48–1.99) for late treatment.

## Discussion

The re-analysis of the UK database shows that time-dependent issues play a crucial role in the question whether NI is associated with mortality in patients with H1N1 influenza. Our main finding is that the treatment of NI (and neither early treatment) is not associated with the daily risk of dying in hospital and there is no confounding by indication. However, we find that the use of NI shortened the length of hospital stay without differences between early and late treatment. This reduction leads to the statistical phenomena that fewer NI-treated patients eventually die in hospital. Therefore, NI intake might indirectly reduces the overall hospital mortality; however, the confidence intervals in Fig 3 indicating no signal for significance. These statements remain unchanged after confounding adjustment and time-dependent propensity score analyses. We also showed that the initiation timing of NI treatment made no difference on the effects on the hospital death and discharge hazards; this is in contrast to previous studies which reported that early initiation (within 2 days after onset) was most beneficial [11, 33].

The main strength of this study is an independent application of advanced statistical methods on data which have been systematically collected during the influenza A/H1N1 pandemic in 2009-2010 in the UK. The application of multistate models allowed us to detect statistical phenomena which remain hidden when using standard methods. Standard Kaplan-Meier curves would overestimate mortality and would not distinguish between hospital death and discharge. And naive length-of-stay comparisons would be biased to the disadvantage of NI since the length-of-stay before NI treatment would be incorrectly added to the NI-treated group.

The main weaknesses of this study are as follows. First, there is limited generalisability to other populations (such as non-hospitalized individuals) and other countries since only hospital data from the UK have been used. Second, the sample size was acceptable but relatively low for studying hospital mortality in more detail (80 patients died in hospital); this might be due to the fact that only 6% were 65 years or older. Third, all potential confounders are measured only at the time of admission but the symptoms might change over time. Therefore, we were not able to update this information in the time-dependent propensity score analysis; it remained constant since admission.

Using the UK data, we observe a different pattern than Muthuri et al [10] who used the larger international meta-analysis with 29.234 patients. In their response letter, they reported an adjusted death hazard ratio for NI-treated patients of 0.54 (95-% CI: 0.47–0.62) and an adjusted discharge hazard ratio of 1.09 (95-% CI: 1.05–1.13) [34]. Based on these numbers (NI-treated patients have a reduced daily risk to die and they are discharged faster), we would expect that the crude overall hospital mortality is remarkably reduced for NI-treated patients. But this is not the case: even more patients who eventually received NI die in hospital compared to patients who did not get NI during hospitalization (9.7% versus 9.2%; see [10]). These findings still require clarification and an in-depth re-analysis of the whole meta-analysis data set would be necessary to explain these discrepancies.

In the international multi-center data base by Muthuri and colleagues, one is faced with further challenges such as potentially different discharge policies or heterogeneity across countries. For instance, a recent systematic review, based on 179 studies from 48 countries, showed that the hospital mortality of influenza A(H1N1)pdm09 ranged from 0% to 52%, with very substantial heterogeneity [35]. This has also consequences for the statistical analysis.

This present re-analysis might have only direct implications for clinicians and policymakers from the UK. The potential beneficial effect of NI on hospitalized patients is rather a reduction of the length of hospital stay than a reduction of mortality. However, this observed effect might be different in other settings.

One important question remains open: the discharge policy of NI-treated and NI-untreated patients could be different. NI-treated patients might continue anti-viral treatment after discharge whereas NI-untreated patients might still remain under (longer) observation in hospital.

In the spirit of the BMJ Tamiflu campaign and the recent call from The Lancet, we believe that it is also necessary to re-analyze further observational studies since the RCTs of NI seem to include only individuals without a real clinical need; and people who might really require the treatment were not included in the RCTs [5]. A report from the Academy of Medical Sciences and the Wellcome Trust and stressed ‘that observational data should not be deemed inferior to randomised trial data when drawing conclusions, as they are often a better measure of real-life events’ [1].

Given the data of observational studies, the survival biases (which are addressed in this analysis) should and can be avoided by appropriate statistical techniques. For improving the data quality of future research, we recommend to follow-up patients beyond discharge and collect daily data on symptoms in order to improve propensity score analyses.

### Glossary

- **confounding by indication:** sicker patients with higher risk of death might be more likely to be given antiviral medications
- **CURB-65 score:** score to predict mortality in infection of any site
- **time-dependent bias:** occurs if a time-dependent exposure or treatment is statistically considered as time-fixed, i.e. as known at the time of baseline
- **time origin / time zero:** the point at which follow-up time starts (in cohort studies often ‘time on study’ or disease onset)
- **delayed entry:** individuals who enter the study later than time origin
- **length bias:** occurs if delayed entry is ignored in the analysis, i.e. assuming that delayed individuals entered already at time origin
- **competing event:** an event which might occur during follow-up and is associated with an altered chance of the event of interest
- **competing risk bias:** occurs if a competing event is handled as censored for risk evaluation, for instance in Kaplan-Meier survival curves
- **multi-state model:** extends the classical two-state survival model (alive-death) and allows more states (such as time-dependent treatment or competing events)
- **cumulative hazard:** a hazard is the instantanous risk of an event; a cumulative hazard plot displays how such a hazard accumulates with time (useful to detect differences in time-dependent treatment groups)
- **landmark method:** method to make risk predictions for several pre-specified time points (landmarks); suitable for multi-state models
- **risk set:** number of individuals at-risk depending on time
- **subdistribution hazard ratio:** compares the cumulative risks with respect to a exposure or treatment in a competing risks setting

## Supporting Information

S1 File Additional results (risksets, multistate model, cumulative hazards, hazard ratios, expected length of stay, multivariate analysis) and statistical code. (TEX)
