# Correction: Neuraminidase Inhibitors and Hospital Mortality in British Patients with H1N1 Influenza A: A Re-Analysis of Observational Data

**DOI:** 10.1371/journal.pone.0175660

**Published:** 2017-04-06

**Authors:** Martin Wolkewitz, Martin Schumacher

S1 File has been uploaded in an incorrect format. Please view the correct S1 File below.

## Supporting information

S1 File Additional results (risksets, multistate model, cumulative hazards, hazard ratios, expected length of stay, multivariate analysis) and statistical code. (PDF)
