# Supplementary material for: Correction: Neuraminidase Inhibitors and Hospital Mortality in British Patients with H1N1 Influenza A: A Re-Analysis of Observational Data
Source: PLoS One. 2017 Apr 6;12(4):e0175660. doi: 10.1371/journal.pone.0175660 (PMC5383344; doi:10.1371/journal.pone.0175660)
Supplement: S1 File — (PDF) [file pone.0175660.s001.pdf]

Neuraminidase inhibitors and hospital mortality in British patients with H1N1 influenza A: a re-analysis of observational data -APPENDIX-

Martin Wolkewitz and Martin Schumacher

Additional results:

- plot of risks sets for NI-untreated and NI-treated
- additional plot of cumulative hospital death and discharge hazards (up to 60 days)
- propensity score analysis
- plot of expected length of stay for NI-untreated and NI-treated depending on time in hospital
- factors associated with the probability of receiving Tamiflu in hospital

Statistical code for:

- imputing missing values for date of influenza onset (SAS code)
- adjustment for potential confounders via covariates (SAS code)
- adjustment for potential confounders via time-dependent propensity score (SAS code)
- calculate the change of length of hospital stay (R code)

Additional results

Fig A: Risksets of NI-treated and NI-untreated patients depending on time from onset. For instance, at day 10 after onset there about 150 patients in hospital without NI treatment and about 250 patients with NI treatment.

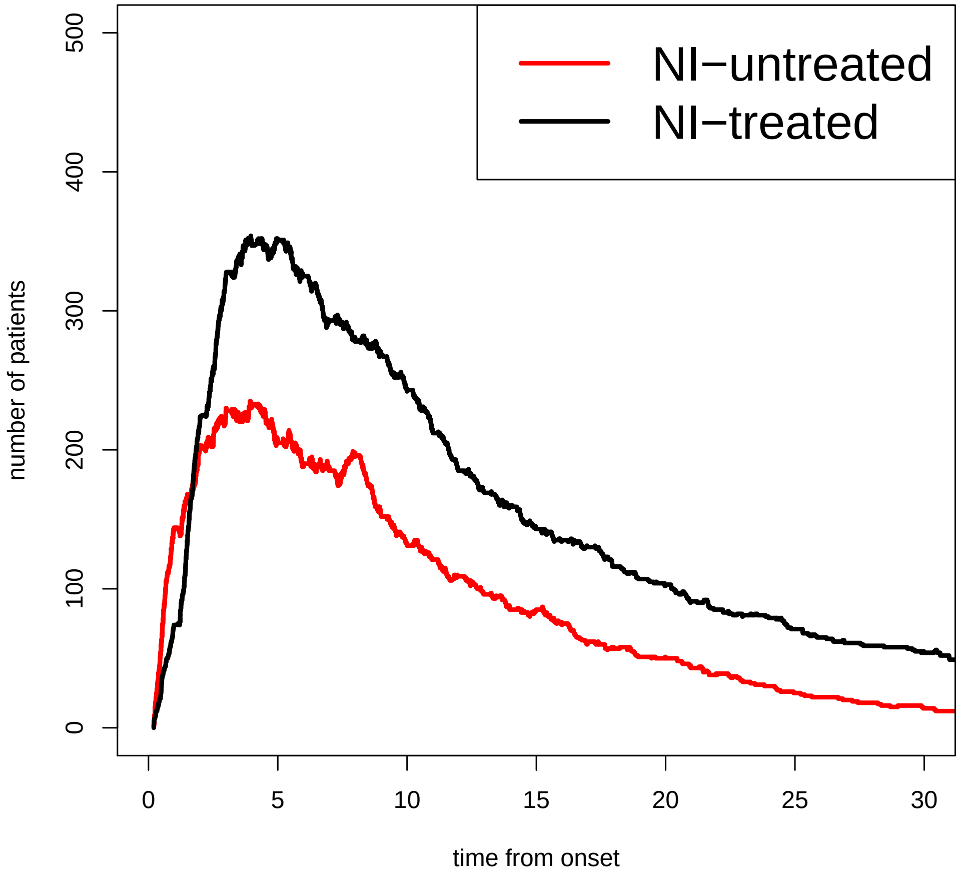

Fig B: Multistate model: Individuals who received Tamiflu (NI) before admission go directly to state 'NI Tamiflu'. Each arrow represents a transition hazard of moving from one state to another.

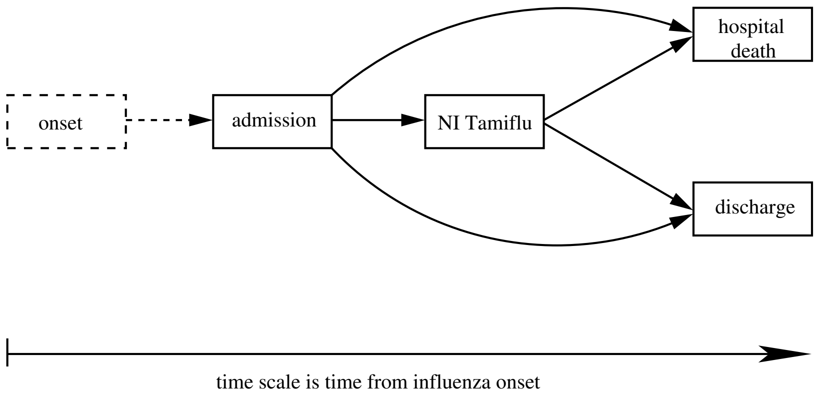

Fig C: Cumulative hospital death and discharge hazards based on the Nelson-Aalen estimator; separately for NI-treatment. Delayed entry (external left-truncation), time-dependency of NI-treatment is accounted for.

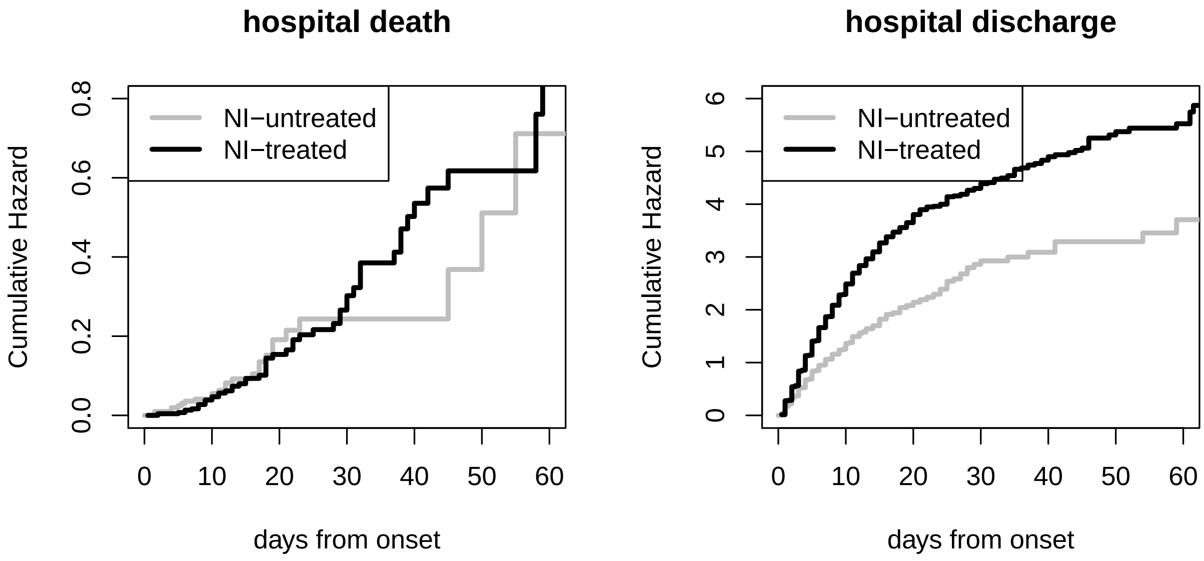

Fig D: Hazard ratios from the Landmark approach. The black dots and lines display the unadjusted (subdistribution) hazard ratios and the gray ones the hazard ratios adjusted via the inverse probability of treatment weighting using the propensity score (PS).

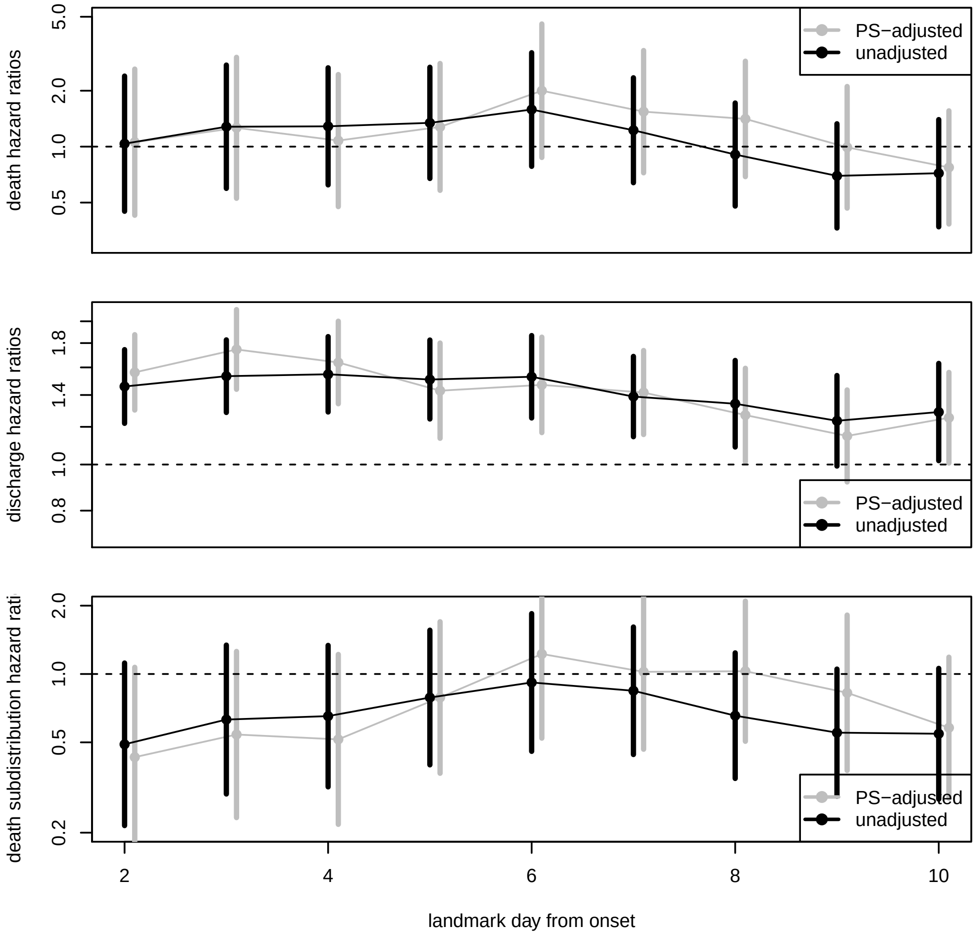

Fig E: Expected length of stay for NI-treated (black) and NI-untreated (red) patients depending on day from admission.

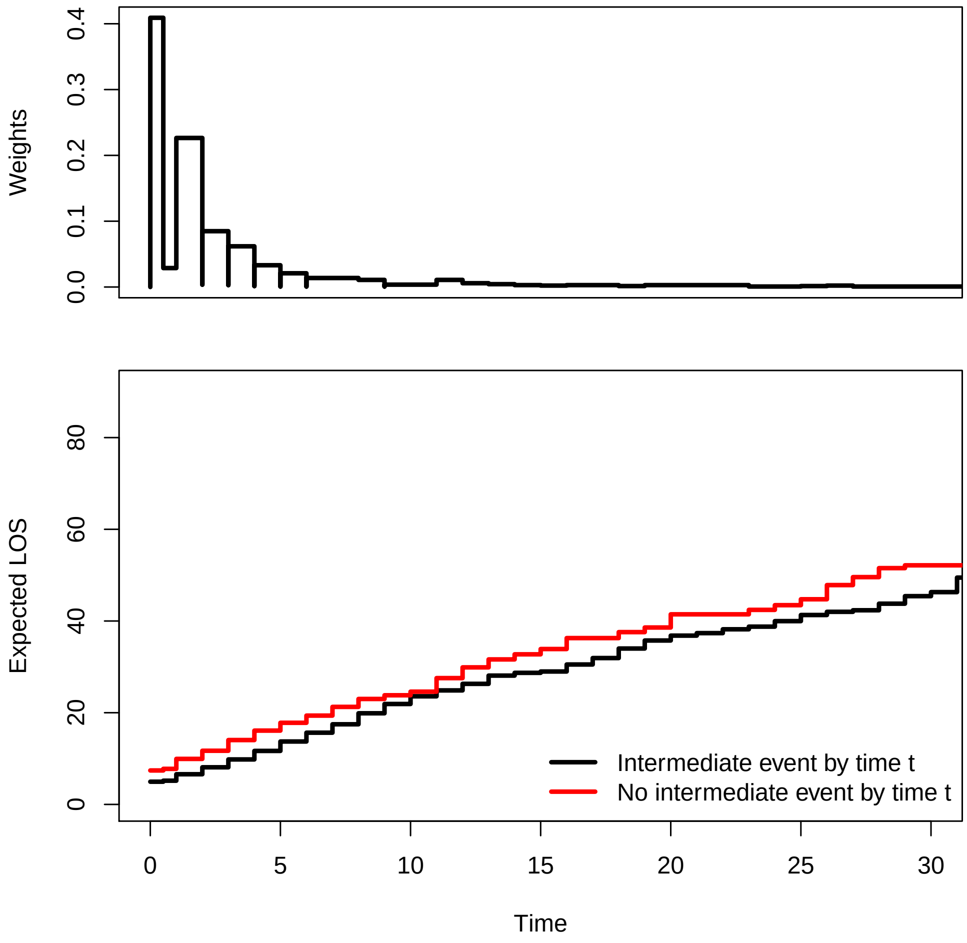

Table 1: Factors associated with the probability of receiving Tamiflu in hospital

We fitted a Fine&Gray model with time from admission (patients who received Tamiflu before admission were excluded) until Tamiflu treatment; patients who are discharged or died without Tamiflu treatment were considered as competing events. Variable selection is made by backward selection and 5% significance level for entering effects. Following factors were included in the regression model: age 0-16 and age 64-95 (age 17-63 as reference), sex, CURB-65 score (categorical), time from onset to admission, asthma, chronic obstructive pulmonary disease, heart disease, other lung diseases, renal disease , liver disease, cerebrovascular disease, diabetes, immunosuppression, hospital id, wave, obese, arthralgia, anorexia, pregnant, dyspnoea, fever, dry cough, diarrhoea, productive cough, coryza, chills, headache, malaise, myalgia, nausea, sore throat, vomiting, confusion, onset date unknown. Results with factors remained after variable selection are shown in table:

**Factor**	**subdistribution hazard ratio (95-% CI)**
age 0-16 years (vs. 17-63 years)	0.77 (0.66-0.91)
male	1.17 (1.01-1.36)
other lung diseases	1.29 (1.13-1.49)
hospital id=39 (vs. reference)	1.14 (0.93-1.39)
hospital id=5 (vs. reference)	0.49 (0.36-0.66)
hospital id=4 (vs. reference)	0.78 (0.65-0.94)
hospital id=3 (vs. reference)	0.77 (0.57-1.04)
hospital id=2 (vs. reference)	1.07 (0.86-1.34)
hospital id=1 (vs. reference)	0.46 (0.33-0.65)
1st wave	1.24 (1.07-1.43)
arthralgia	1.32 (1.03-1.68)
pregnant	0.79 (0.65-0.95)
fever	1.21 (1.02-1.44)
dry cough	1.25 (1.07-1.46)
productive cough	1.19 (1.02-1.38)
headache	1.31 (1.13-1.52)

Table 2: Multivariate analysis regarding the hazard to die in hospital

Time-dependent Cox proportional hazard model for hospital death hazard. Variable selection is made with the same variables as above by backward selection and 5% significance level for entering effects (Neuraminidase inhibitors always included).

Factor	hazard ratio (95-% CI)
Neuraminidase inhibitors	1.04 (0.62-1.75)
CURB-65 score 3/4 (vs. score 0)	4.57 (1.67-12.5)
CURB-65 score 2 (vs. score 0)	2.36 (0.99-5.58)
CURB-65 score 1 (vs. score 0)	1.97 (0.85-4.56)
dry cough	0.46 (0.25-0.84)
diarrhoea	1.88 (1.03-3.44)
myalgia	0.36 (0.13-0.99)

Table 3: Early and late NI treatment regarding hospital death

Some model as above, now differentiating early ($\leq$ 2 days after onset) and late (> 2 days after onset) NI treatment versus no NI-treatment.

Factor	hazard ratio (95-% CI)
NI ($\leq$ 2 days after onset)	0.96 (0.55-1.67)
NI (> 2 days after onset)	1.16 (0.59-2.29)
CURB-65 score 3/4 (vs. score 0)	4.78 (1.77-13.0)
CURB-65 score 2 (vs. score 0)	2.62 (1.12-6.15)
CURB-65 score 1 (vs. score 0)	2.01 (0.87-4.61)
dry cough	0.46 (0.25-0.85)

Table 4: Multivariate analysis regarding the hazard to be discharged from the hospital

Time-dependent Cox proportional hazard model for hospital discharge hazard. Variable selection is made with the same variables as above by backward selection and 5% significance level for entering effects (Neuraminidase inhibitors always included).

Factor	hazard ratio (95-% CI)
Neuraminidase inhibitors	2.00 (1.73-2.31)
age 0-16 years (vs. 17-63 years)	1.47 (1.26-1.70)
CURB-65 score 3/4 (vs. score 0)	0.55 (0.40-0.77)
CURB-65 score 2 (vs. score 0)	0.79 (0.67-0.94)
CURB-65 score 1 (vs. score 0)	0.74 (0.63-0.86)
>7 days between onset and admission (vs. 0-2 days)	0.86 (0.71-1.04)
5-7 days between onset and admission (vs. 0-2 days)	0.78 (0.65-0.94)
3-4 days between onset and admission (vs. 0-2 days)	0.84 (0.72-0.99)
asthma	1.21 (1.04-1.41)
immunosuppression	0.67 (0.46-0.99)
dyspnoea	0.74 (0.64-0.84)
coryza	1.21 (1.02-1.43)
headache	1.49 (1.28-1.74)
sore throat	1.26 (1.08-1.46)
vomiting	1.28 (1.12-1.48)

Table 5: Early and late NI treatment regarding hospital discharge

Some model as above, now differentiating early ($\leq$ 2 days after onset) and late (> 2 days after onset) NI treatment versus no NI-treatment.

Factor	hazard ratio (95-% CI)
NI ($\leq$ 2 days after onset)	1.84 (1.58-2.14)
NI (> 2 days after onset)	1.72 (1.48-1.99)
age 0-16 years (vs. 17-63 years)	1.41 (1.24-1.61)
CURB-65 score 3/4 (vs. score 0)	0.63 (0.46-0.86)
CURB-65 score 2 (vs. score 0)	0.84 (0.72-0.99)
CURB-65 score 1 (vs. score 0)	0.80 (0.70-0.91)
asthma	1.15 (1.01-1.31)
dyspnoea	0.79 (0.70-0.89)
headache	1.39 (1.22-1.60)
sore throat	1.19 (1.04-1.36)
vomiting	1.18 (1.10-1.33)
onset unknown	0.80 (0.70-0.91)

Statistical code in SAS

Imputing missing values for date of influenza onset (SAS code)

```
proc mi data=redux_mi out=imp round=1 MINIMUM=0 NIMPUTE=1 SEED=1234567;
class age_0_16 age_64_95
sex CURB-65_score_cat my_asthma my_copd my_heart_ds my_other_lung_ds
my_renal_ds my_liver_ds
my_cerebrovascular_ds my_diabetes  my_immunosuppression my_hospital_id
wave my_obese my_arthralgia my_anorexia my_pregnant
my_dyspnoea my_fever my_dry_cough my_diarrhoea my_productive_cough
my_coryza my_chills my_headache my_malaise my_myalgia my_nausea
my_sore_throat my_vomiting my_confusion ;

var  my_length_of_stay age_0_16 age_64_95
sex CURB-65_score_cat my_asthma my_copd my_heart_ds my_other_lung_ds
my_renal_ds my_liver_ds
my_cerebrovascular_ds my_diabetes  my_immunosuppression my_hospital_id
wave my_obese my_arthralgia my_anorexia my_pregnant
my_dyspnoea my_fever my_dry_cough my_diarrhoea my_productive_cough
my_coryza my_chills my_headache my_malaise my_myalgia my_nausea
my_sore_throat my_vomiting my_confusion diff_admission_onset;

monotone reg(diff_admission_onset=my_length_of_stay age_0_16 age_64_95
sex CURB-65_score_cat my_asthma my_copd my_heart_ds my_other_lung_ds
my_renal_ds my_liver_ds
my_cerebrovascular_ds my_diabetes  my_immunosuppression my_hospital_id
wave my_obese my_arthralgia my_anorexia my_pregnant
my_dyspnoea my_fever my_dry_cough my_diarrhoea my_productive_cough
my_coryza my_chills my_headache my_malaise my_myalgia my_nausea
my_sore_throat my_vomiting my_confusion);
run;

proc sort data=f;by patid;run;
proc sort data=imp;by patid;run;
data f_imp;
merge f imp;
by patid;
onset_date=admission_date-diff_admission_onset;
run;
```

Adjustment for potential confounders via covariates (SAS code)

```
*the data ta.mvna uses the start-stop notation for the corresponding multi-state
model explained in the main text;

title 'death adjusted (all) adm';
proc phreg data=ta.mvna;
class  sex CURB-65_score_cat my_admission_cat/descending;
strata my_hospital_id;
model (entry_clos,exit_clos)*to(1,2,4)=from age_0_16 age_64_95 sex
CURB-65_score_cat my_admission_cat
```

```
my_asthma my_copd my_heart_ds my_other_lung_ds my_renal_ds my_liver_ds
my_cerebrovascular_ds my_diabetes  my_immunosuppression  wave
my_obese my_arthralgia my_anorexia my_pregnant
my_dyspnoea my_fever my_dry_cough my_diarrhoea my_productive_cough
my_coryza my_chills my_headache my_malaise my_myalgia my_nausea
my_sore_throat my_vomiting my_confusion onset_unknown /
rl INCLUDE=1 selection=backward slstay=0.05 ;
run;


title 'discharge adjusted (all) adm';
proc phreg data=ta.mvna;
class  sex CURB-65_score_cat my_admission_cat/descending;
strata my_hospital_id;
model (entry_clos,exit_clos)*to(1,3,5)=from age_0_16 age_64_95
sex CURB-65_score_cat my_admission_cat
my_asthma my_copd my_heart_ds my_other_lung_ds my_renal_ds my_liver_ds
my_cerebrovascular_ds my_diabetes  my_immunosuppression  wave
my_obese my_arthralgia my_anorexia my_pregnant
my_dyspnoea my_fever my_dry_cough my_diarrhoea my_productive_cough
my_coryza my_chills my_headache my_malaise my_myalgia my_nausea
my_sore_throat my_vomiting my_confusion onset_unknown /
rl INCLUDE=1 selection=backward slstay=0.05 ;
run;
```

Adjustment for potential confounders via time-dependent propensity score (SAS code)

```
libname ta "/home/wolke/SAS/DFG/progs/Tamiflu/Real/";
%include "/home/wolke/SAS/MACROS/quint.sas";

%macro land (lm=,range=);
data ps&lm._;
set g;
where my_admission<=&lm. and my_discharge>&lm. and tamiflu_before=0;
if .<my_tamiflu<=&lm..5 then tamiflu_&lm=1;
else tamiflu_&lm=0;
run;

proc logistic descending data = ps&lm._;
class my_hospital_id sex CURB-65_score_cat my_admission_cat/descending;
   model  tamiflu_&lm= age_0_16 age_64_95 sex CURB-65_score_cat my_admission_cat
my_asthma my_copd my_heart_ds my_other_lung_ds my_renal_ds my_liver_ds
my_cerebrovascular_ds my_diabetes  my_immunosuppression my_hospital_id wave
my_obese my_arthralgia my_anorexia my_pregnant
my_dyspnoea my_fever my_dry_cough my_diarrhoea my_productive_cough
my_coryza my_chills my_headache my_malaise my_myalgia my_nausea
my_sore_throat my_vomiting my_confusion onset_unknown
   /lackfit outroc = ps_r ;
   output out = ps&lm._  XBETA=ps_xb&lm STDXBETA= ps_sdxb&lm PREDICTED = ps_pred&lm;
run;

proc sort data=g;by my_id;run;
proc sort data=ps&lm._;by my_id;run;
data ps&lm;
merge g(where=(my_admission<=&lm. and my_discharge>&lm.)) ps&lm._ ;
```

```
by my_id;
run;

%quint(ps&lm.,ps_pred&lm.,ps_pred&lm._q);

data ps&lm ;
set ps&lm ;
if ps_pred&lm.=. then do;ps_pred&lm.=0.9-0.1*ranuni(1); tamiflu_&lm=1;end;
if my_discharge<=&lm+&range then do;my_discharge_&lm.=my_discharge;
my_death_&lm.=my_death;end;
else do my_discharge_&lm.=&lm+&range;my_death_&lm.=0;end;
if my_death_&lm=0 then my_discharge_fg_&lm=&lm+&range;
else my_discharge_fg_&lm=my_discharge_&lm.;
if tamiflu_&lm=1 then w=1/ps_pred&lm;else  w=1/(1-ps_pred&lm);
entry_lm=&lm;
run;

title 'death';
ods trace off;
ods output ParameterEstimates=de_&lm;
proc phreg data=ps&lm;
model (entry_lm,my_discharge_&lm.)*my_death_&lm.(0)= tamiflu_&lm / rl;
run;
title 'death adjusted';
ods trace off;
ods output ParameterEstimates=deadj_&lm;
proc phreg data=ps&lm  covs(aggregate);
model (entry_lm,my_discharge_&lm.)*my_death_&lm.(0)= tamiflu_&lm   / rl;
weight w;
id patid;
run;

title 'discharge';
ods output ParameterEstimates=dis_&lm;;
proc phreg data=ps&lm;
model (entry_lm,my_discharge_&lm.)*my_death_&lm.(1)= tamiflu_&lm / rl;
run;

title 'discharge-adjusted';
ods output ParameterEstimates=disadj_&lm;;
proc phreg data=ps&lm  covs(aggregate);
model (entry_lm,my_discharge_&lm.)*my_death_&lm.(1)= tamiflu_&lm/rl;
weight w;
id patid;
run;

title 'death f&g';
ods trace off;
ods output ParameterEstimates=defg_&lm;;
proc phreg data=ps&lm;
model (entry_lm,my_discharge_fg_&lm.)*my_death_&lm.(0)= tamiflu_&lm/rl;
run;

title 'death f&g adjusted';
ods trace off;
ods output ParameterEstimates=defgadj_&lm;;
proc phreg data=ps&lm  covs(aggregate);
model (entry_lm,my_discharge_fg_&lm.)*my_death_&lm.(0)= tamiflu_&lm/rl;
weight w;
id patid;
run;
```

```
data pe_&lm;
set de_&lm deadj_&lm  dis_&lm disadj_&lm defg_&lm defgadj_&lm  ;
day=&lm;
where parameter="tamiflu_&lm";
if _n_=1 then out="de     ";
else if _n_=2 then out="deadj ";
else if _n_=3 then out="dis   ";
else if _n_=4 then out="disadj";
else if _n_=5 then out="fg    ";
else if _n_=6 then out="fgadj";
run;

ods output crosslist=cl&lm;
proc freq  data=ps&lm;
tables tamiflu_&lm*my_death_&lm./crosslist;
run;

data cl&lm.;
set cl&lm.(where=(tamiflu_&lm. ne .));
tamiflu=tamiflu_&lm.;
my_death=my_death_&lm.;
day=&lm;
keep day tamiflu my_death frequency rowpercent ;
run;
%mend;

%macro pool (pool=);
data g;
set ta.g;
rauschen=0.8*ranuni(&pool.)+0.1;
my_admission=my_admission+rauschen;
my_tamiflu=my_tamiflu+rauschen;
my_discharge=my_discharge+rauschen;
tamiflu_actual=tamiflu_actual+rauschen;
run;

%land (lm=2,range=20);
%land (lm=3,range=20);
%land (lm=4,range=20);
%land (lm=5,range=20);
%land (lm=6,range=20);
%land (lm=7,range=20);
%land (lm=8,range=20);
%land (lm=9,range=20);
%land (lm=10,range=20);

data pe_pool&pool.;
set pe_2-pe_10;
pool=&pool;
run;

data cl_pool&pool.;
set cl2-cl10;
pool=&pool;
run;
%mend;

%pool (pool=1);
%pool (pool=2);
%pool (pool=3);
```

```
%pool (pool=4);
%pool (pool=5);
%pool (pool=6);
%pool (pool=7);
%pool (pool=8);
%pool (pool=9);
%pool (pool=10);

data pe_ps_pool_p;
set pe_pool1-pe_pool10;
run;

proc means data=pe_ps_pool_p mean;
class out day ;
var estimate stderr;
output out=pe_ps_pool ;
run;

data pe_ps_pool;
set pe_ps_pool;
where _type_=3 and _stat_="MEAN";
hr=exp(estimate);
upper=exp(estimate+1.96*stderr);
lower=exp(estimate-1.96*stderr);
run;

data cl_pool_c;
set cl_pool1-cl_pool10;
run;

proc means data=cl_pool_c mean;
class tamiflu day ;
var rowpercent;
where my_death=1;
output out=cl_ps_pool mean=mean;
run;

data cl_ps_pool;
set cl_ps_pool;
where _type_=3;
run;
```

Calculate the change of length of hospital stay (R code)

```
library(etm)
mv_<-read.csv('mvna_real.csv')

## function that performs the bootstrap
## nboot: number of bootstrap samples. Other arguments are as in etm()
set.seed(22)
boot.clos <- function(data, state.names, tra, cens.name, s = 0, nboot) {
  res <- double(nboot)
  for (i in seq_len(nboot)) {
    index <- sample(unique(data$id), replace = TRUE)
    inds <- new.id <- NULL
    for (j in seq_along(index)){
      ind <- which(data$id == index[j])
```

```
      new.id <- c(new.id, rep(j, length(ind)))
      inds <- c(inds, ind)
    }
    dboot <- cbind(data[inds, ], new.id)
    dboot[, which(names(dboot) == "id")]
    dboot$id <- dboot$new.id
    tr.prob <- etm(dboot, state.names, tra, cens.name, s, cova = FALSE)
    res[i] <- etm::clos(tr.prob)$e.phi
  }
  res
}
## bootstrap

mv<-subset(mv_,select=c(entry_clos,exit_clos,from,to))
mv$id<-mv_$my_id
names(mv)<-c("entry","exit","from","to","id" )
mv$to<-ifelse(mv$to %in% c(2,3,4,5),2,mv$to)

tra <- matrix(ncol=3,nrow=3,FALSE)
tra[1, c(2,3)] <- TRUE
tra[2, 3] <- TRUE
tra

pr<-etm(mv,c("0","1","2"),tra,NULL,s=0)
los<-clos(pr,aw=T)
los
se <- sqrt(var(boot.clos(mv, c("0","1","2"), tra, NULL, 0,
                              nboot = 1000)))
pdf("los.pdf")
plot(los,xlim=c(0,30))
dev.off()

low<-los$e.phi-1.96*se
up<-los$e.phi+1.96*se
clos<-cbind(round(los$e.phi,digits=2),round(low,digits=2),round(up,digits=2))
clos
```
